# Gaze stabilization in mantis shrimp in response to angled stimuli

**DOI:** 10.1007/s00359-019-01341-5

**Published:** 2019-05-16

**Authors:** Ilse M. Daly, Martin J. How, Julian C. Partridge, Nicholas W. Roberts

**Affiliations:** 1 School of Biological Sciences, University of Bristol, Tyndall Avenue, Bristol, BS8 1TQ UK; 2 Oceans Institute, University of Western Australia, 35 Stirling Highway, (M470), Crawley, WA 6009 Australia

**Keywords:** Stomatopod, Gaze stabilisation, Optokinesis, Motion detection, Eye movements

## Abstract

Gaze stabilization is a fundamental aspect of vision and almost all animals shift their eyes to compensate for any self-movement relative to the external environment. When it comes to mantis shrimp, however, the situation becomes complicated due to the complexity of their visual system and their range of eye movements. The stalked eyes of mantis shrimp can independently move left and right, and up and down, whilst simultaneously rotating about the axis of the eye stalks. Despite the large range of rotational freedom, mantis shrimp nevertheless show a stereotypical gaze stabilization response to horizontal motion of a wide-field, high-contrast stimulus. This response is often accompanied by pitch (up-down) and torsion (about the eye stalk) rotations which, surprisingly, have no effect on the performance of yaw (side-to-side) gaze stabilization. This unusual feature of mantis shrimp vision suggests that their neural circuitry for detecting motion is radially symmetric and immune to the confounding effects of torsional self-motion. In this work, we reinforce this finding, demonstrating that the yaw gaze stabilization response of the mantis shrimp is robust to the ambiguous motion cues arising from the motion of striped visual gratings in which the angle of a grating is offset from its direction of travel.

## Introduction

**Fig. 1:**
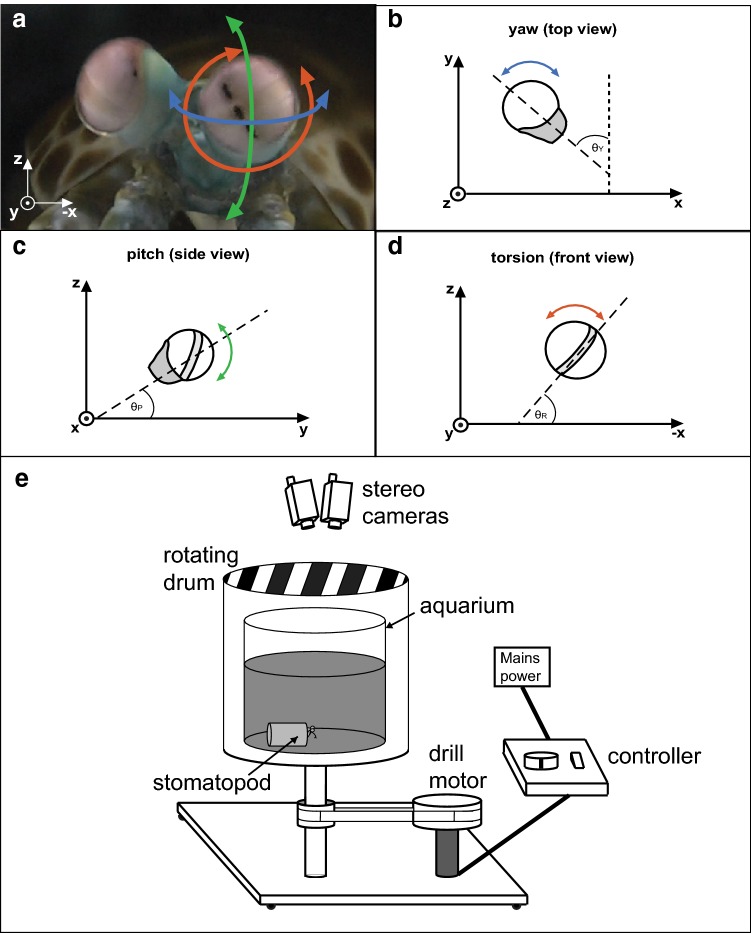
**a** The three degrees of rotational freedom in the stomatopod eye; yaw (side-to-side, blue arrow), pitch (up-down, green) and torsion (rotation about the eye axis, red). **b** We define ‘yaw’ as the rotation about the *z*-axis, in the *x*–*y* plane of the real-world coordinate system. Similarly, **c** ‘pitch’ is defined as the rotation about the *x*-axis, in the *y*–*z* plane and **d** torsion as the rotation about the *y*-axis, in the *x*–*z* plane of the real world. Calculation of pitch and torsion assumes alignment with the real-world axis, and, therefore, require compensation for the yaw pose. The pitch pose is independent of the torsion angle; it is the elevation of the eye regardless of its torsional pose. **e** The rotating drum and aquarium set-up including the positioning of the animal and the stereo cameras used during the experiment. Figure adapted from Fig. 2 in (Daly et al. 2017)

The complexity of the mantis shrimp visual system has been well documented. With as many as 16 anatomically diverse photoreceptor classes, stomatopods have up to 12-channel colour vision, alongside both linear and circular polarization vision (Marshall 1988; Marshall et al. 1991a, b, 2007; Chiou et al. 2008; Roberts et al. 2009; Thoen et al. 2014; How et al. 2014a, b). Their compound eyes, which are of the apposition type, develop into three distinct functional parts: dorsal and ventral hemispheres separated across the eye’s equator by a midband comprising 2, 4 or 6 rows (depending on species) of enlarged ommatidia (Exner 1891; Marshall et al. 1991a). The photoreceptor classes mediating colour vision are restricted to the midband, effectively resulting in 1D colour vision requiring scanning eye movements and the serial acquisition of visual information for colour determination (Marshall et al. 1991a; Thoen et al. 2014). Additionally, the axes of the ommatidia in the hemispheres are skewed towards the equator, resulting in the acute zone of the eye having an effective field of view of approximately 15° (Schiff 1963; Horridge 1978; Manning et al. 1984; Marshall and Land 1993a, b; Marshall et al. 2007; Marshall and Arikawa 2014). Probably as a consequence of their unique anatomy, stomatopod eyes have unprecedented freedom of movement with more than 90° angular range in all three rotational degrees of freedom, with rotations about the base of the eyestalk causing: pitch (up-down), yaw (side-to-side), and torsion (rotation about the long axis of the eye stalk) (Fig. 1a, d). This range of rotational freedom allows stomatopods to make ‘scans’ of the visual scene, which are thought to be used to gain visual information (especially colour and polarization) from an entire scene and not just the narrow strip that is viewed at any one time by the specialized midband ommatidia (Land et al. 1990). The eyes are capable of independent movement (Jones 1994), although their movements may become coupled during certain visual tasks such as startle saccades (Daly et al. 2017). In addition, they have been shown to exhibit gaze stabilization responses in the yaw and pitch (Land et al. 1990; Cronin et al. 1991), but not in the torsional, degrees of rotational freedom (Daly et al. 2018).

The gaze stabilization reflex is an inherent trait across almost all animal taxa. Whilst having mobile eyes presents an animal with distinct adaptive advantages in areas such as locomotion and prey capture (Land 1999), overcoming the degrading effects on the retinal image due to the resulting motion blur presents a significant challenge. Not only is it more difficult to detect an object, either stationary or in motion, relative to its background in a blurred image than in a spatio-temporally stabilized one, but motion blur also disrupts an animal’s ability to infer information from optic flow or motion parallax (Nalbach 1990; Land 1999). Furthermore, an animal’s egocentric coordinate system can become misaligned with real-world coordinates unless there is adequate visual compensation for rotational and translational movements of the body and/or eyes, potentially compromising body posture and equilibrium (Nalbach et al. 1989; Nalbach 1990). Animals counteract these degrading visual effects by making compensatory movements with their eyes, head or body (depending on their individual anatomy) to reduce movement of the retinal image (Nalbach 1990). This is known as gaze stabilization, and is common to both vertebrates and arthropods (Horridge and Sandeman 1964; Sandeman et al. 1975; Wiersma et al. 1982; Land 1999; Daly et al. 2018).

These compensatory movements may be mediated by the vestibular system to produce the vestibular-ocular reflex (VOR), or just purely by the visual system, resulting in optokinetic (OKR) or optomotor (OMR) responses. The OKR consists of a repetitive series of eye movements with a slow and a fast phase, known as the optokinetic nystagmus (Horridge and Sandeman 1964; Tauber and Atkin 1967; Cronin et al. 1991; Land 1999; Fritsches and Marshall 2002; Land 2014). During the slow phase, the eye typically performs a rotation in the same direction and at approximately the same speed as the movement of the visual scene followed, at intervals, by the fast phase; a rapid counter rotation, which ‘flicks’ the eye back to the approximate starting position (Horridge and Sandeman 1964; Cronin et al. 1991; Land 2014). The slow phase of optokinetic nystagmus largely ‘fixes’ an image on the retina and is seen in animals both with and without a fovea or acute zone. The OMR is similar to the OKR, but involves movement of the entire body not just the eyes (Horridge and Sandeman 1964).

While stomatopods show stereotypical OKR in response to the horizontal translation of the visual scene, the yaw (Fig. 1b) eye movements are often accompanied by pitch (Fig. 1c) and torsion rotations (Fig. 1d). Surprisingly, eye rotations about the axis of the eye stalk do not affect yaw gaze stabilization performance (Daly et al. 2018). This is unexpected because the torsional rotations act to shift the apparent direction of motion of an object, as projected onto the retina, even if the object is following a steady course. For instance, a stimulus moving horizontally when the stomatopod eye is held such that the midband is horizontal (*θ*_T_ = 0°), will appear to be moving vertically in the reference frame of the eye when the midband is rotated to a vertical position (*θ*_T_ = 90°). However, as the consistency in gaze stabilization demonstrates, eye rotations about the axis of the eye stalk (torsion) do not affect the ability of stomatopods to track object motion with accuracy. Stomatopods do not, however, exhibit a torsional gaze stabilization response (Daly et al. 2018). These findings strongly suggest the neural wide-field motion detection network in the stomatopod visual system may follow a radially symmetric organization that allows yaw and pitch tracking regardless of eye torsion (Cronin et al. 1991; Daly et al. 2018).

In this work, we follow up these previously reported findings by testing the extent to which the stomatopod gaze stabilization response is robust to ambiguous motion cues. In previous investigations into the OKR, the stimulus comprised a grating of vertical high-contrast stripes, which create an unambiguous motion cue in the perpendicular direction to the long axis of the stripes. Here, we present a more ambiguous motion cue by altering the orientation of the grating by either 10°or 20° from the vertical (0°). We ask: (1) is the gaze stabilization performance in the yaw degree of rotational freedom affected by the orientation of the grating? (2) Is the torsional pose of the eye affected by the orientation of the grating? (3) Does the ambiguity of the angular offset between the direction of motion and the orientation of the grating require a greater level of torsional stability? (4) Are stomatopods susceptible to visual illusions caused by an angular offset between the direction of motion and the orientation of the grating?

## Methods

### Stomatopods

Stomatopod ‘mantis shrimps’ of the species *Odontodactylus scyllarus* of varying sizes (carapace lengths between 9 and 15 cm) were purchased from commercial sources (Tropical Marine Centre, Bristol, UK) and were maintained in the aquatic facility at the University of Bristol, UK under a 12:12 h light cycle. All specimens were originally captured from the natural environment and were held in the laboratory for less than 3 months prior to the experiment.

Experiments were conducted under University of Bristol permit UIN/15/050.

### Experimental set-up

Yaw optokinesis was elicited in individual stomatopods by the horizontal motion of a black and white grating on the inner face of a rotating drum following the method of Daly et al. (2017), and as shown in Fig. 1e. Six *Odontodactylus scyllarus* were placed individually in an artificial ‘burrow’ (a horizontally orientated, 3 cm diameter 8 cm long opaque plastic tube) in a 20 cm radius, vertically orientated, cylindrical transparent acrylic (Perspex™) aquarium fixed in the centre of a larger co-axial rotating Perspex™ cylinder (diameter 30 cm, height 40 cm). The central cylinder was filled to a depth of 15 cm with seawater from the animal’s home aquarium. The external rotating cylinder was not filled with water and was free to spin in the horizontal plane about the vertical z-axis, driven by an electric motor (970D, Como Drills, Kent, UK) at an angular speed of 11.63 ± 0.44°s^−1^ (mean ± standard deviation) in either the clockwise or anticlockwise direction. The visual stimulus was provided by a grating comprising 24 pairs of black and white stripes of equal widths (1.96 cm) printed on A3 paper and attached to the inner side of the drum, with each pair subtending a visual angle of 15° from the position of the head of the experimental animal. The acceptance angle of the ommatidia in the visual streak (acute zone) of *O. scyllarus* is 0.6° and the inter-ommatidial angle is 0.5° (Marshall and Land 1993b) indicating that the grating will be easily resolved. The absolute irradiance spectra of the black (I_B_(λ)) and white (I_W_(λ)) regions of the grating under the illumination conditions of the experiment were obtained using a photospectrometer (QE6500, Ocean Optics, FL, USA) and SpectraSuite (Ocean Optics, FL, USA) with an optical fibre (QP200-2-UV–VIS, Ocean Optics, FL, USA) and a lens tube (acceptance angle 5°) located at the same position as the animal. These absolute irradiance spectra were used to calculate the Michelson contrast at each wavelength, C_M_(λ), of the grating using the relation

1 $$C_{\text{M}} \left( \lambda \right) = \frac{{I_{\text{W}} \left( \lambda \right) - I_{\text{B}} \left( \lambda \right)}}{{I_{\text{W}} \left( \lambda \right) + I_{\text{B}} \left( \lambda \right)}}.$$

**Fig. 2:**
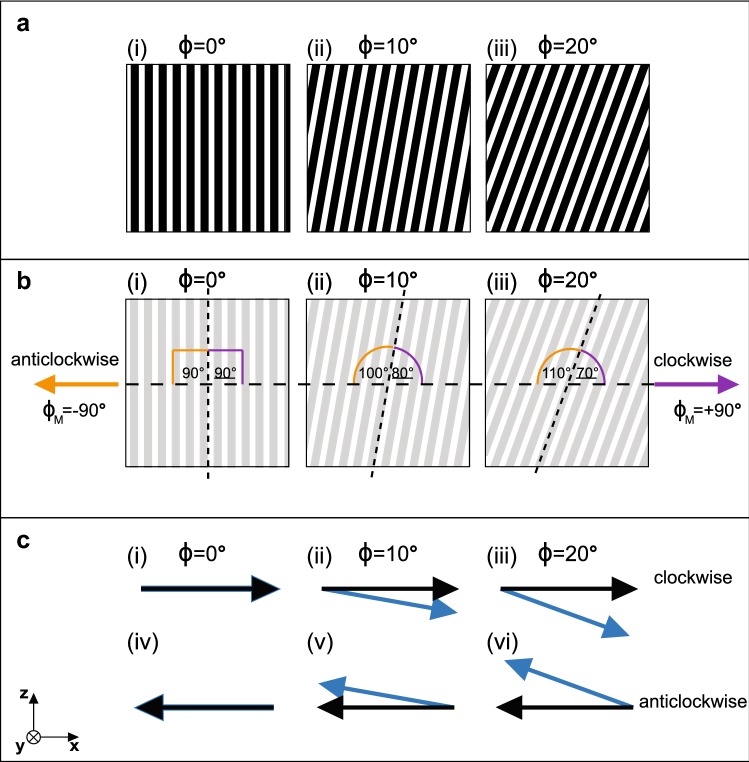
**a** Three orientations of grating (*φ*) were presented to each animal: (i) 0°, (ii) 10°, and (iii) 20° in both directions in the horizontal plane; **b** (i–iii) clockwise (purple) and anticlockwise (orange). The angular offset (*δ*) between the stripe axis and motion direction (*φ*_M_) is dependent on the direction of rotation (clockwise *φ*_M_ = 90°; clockwise *φ*_M_ = − 90°) and on the orientation of the grating; the values of δ for each stripe orientation during clockwise rotation are shown to the right (underlined; purple angle measure) and during anticlockwise rotation to the left (not underlined; orange angle measure). **c** The direction of the apparent motion in the ‘real-world’ coordinate system (calculated using the model of How and Zanker 2014) for each grating orientation during (i–iii) clockwise and (iv–vi) anticlockwise rotation at each grating orientation is represented by the blue arrow. The actual direction of motion (horizontal in all cases) is represented by the black arrow. When *φ* = 0°, the apparent motion of the grating is purely horizontal, whilst when *φ* = 10° or *φ* = 20°, the apparent motion has a vertical component

The average Michelson contrast was 0.94 in the 420–700 nm range of the spectrum, indicating that the black and white grating had a high intensity contrast. 420 nm was chosen as the lower cut-off for this calculation due to the experimental light source having no UV component. Three versions of the high contrast gratings were used, with the angle (*φ*) of the stripes offset from the vertical by 0°, 10° or 20° in each treatment (Fig. 2a). Each animal was presented with the three versions of the grating in two instances, each rotating for 45 s in both the clockwise and anticlockwise direction. The stimuli, and direction of rotation, were presented in a pseudo-randomised order.

The three-dimensional movements (yaw, pitch and torsion) of the two eyes were recorded using two video camcorders (Panasonic HC-X900, Osaka, Japan) calibrated to form a stereoscopic pair and tracked in each frame (50fps) using MATLAB (2015b, Mathworks, Massachusetts, USA). We represent rotations with Tait–Bryan angles calculated in the laboratory reference frame using an intrinsic coordinate system in the order [*z*–*x′*–*y*′′]. We define yaw, described by the angle θ_Y_, to be the rotation of the eye in the *x*–*y* plane about the *z*-axis of the ‘real-world’ coordinate system and causing side-to-side movement of the eye (Fig. 1b). We define pitch (*θ*_P_) as the rotation of the eye in the *y*–*z* plane about the *x*-axis, causing up-down eye movements (Fig. 1c), and torsion (*θ*_T_) as roll rotation in the *x*–*z* plane about the *y*-axis (Fig. 1d). The *x*-*, y*- and *z*- Cartesian coordinates of a set of tracking markers affixed to the eye stalk of the animal were used to calculate *θ*_Y_, *θ*_P_ and *θ*_T_ (Daly, in submission).

### Yaw Gaze stabilization analysis

The rotation of the drum causes wide-field horizontal motion of the visual field (Fig. 2b), which elicits a stereotypical OKR-type gaze stabilisation response involving yaw rotation of the eye (Cronin et al. 1991; Daly et al. 2017). During optokinesis, an eye tracks the movement of the visual field before performing a rapid counter rotation to ‘reset’ its position. The performance of an eye when stabilising its gaze can be quantified using the relative velocity ratio (previously termed ‘gain’ (Land et al. 1990; Cronin et al. 1991)), which is the ratio between the angular velocity of the drum and the angular velocity of the eye in the yaw (side-to-side) degree of freedom. Since the drum is only free to rotate about the *z*-axis, the angular speed of the drum will be purely in the *x*–*y* yaw plane. The relative velocity ratio in the yaw degree of freedom, S_Y_, is given by

2 $$S_{\text{y}} = \frac{{{\text{eye}}\_{\text{angular}}\_{\text{velocity}}}}{{{\text{drum}}\_{\text{angular}}\_{\text{velocity}}}}.$$

If the angular velocity of the drum is exactly matched by the yaw rotation of the eyes, *S*_Y_ = 1, corresponding to perfect gaze stabilisation. In reality, due to the delay caused by the finite response time of the animal visual system and because some retinal slippage is required for a closed-loop feedback system controlling the eyes’ tracking movements, *S*_Y_ is usually < 1. If *S*_Y_ > 1, the angular velocity of the eye exceeds that of the drum; if the eye is stationary, *S*_Y_ = 0. If the eye rotates in the opposite direction to the drum, as it does during the fast reset phase of optokinetic nystagmus, then *S*_Y_ < 0. In some of the following statistical analyses S_Y_ is calculated only during the smooth tracking phase of the optokinetic nystagmus, in which the eye rotates in the same direction of the drum, such that *S*_Y _≥ 0. Several figures include values of S_Y_ during both slow (tracking) and fast (reset) phases to demonstrate the overall trend in yaw rotation of the eyes during optokinesis.

The angular velocity of the eyes used to calculate S_Y_ is calculated as the change in the angular (yaw) pose between successive frames of the video, rather than manually selecting regions of the responses. This is to avoid human error in determining when a movement began or ended, and to ensure that all available data is used. Similarly, this ensures that equal numbers of data are used from each eye and each individual animal.

### Apparent motion analysis

The rotating gratings create a wide-field motion stimulus when the drum rotates in the horizontal plane. For the tilted gratings (*φ* = 10° or *φ* = 20°), the long axes of the stripes are non-orthogonal to the direction of motion, while the vertical stripes of the un-tilted grating (*φ* = 0°) are perpendicular to it (Fig. 2b(i)–(iii)). The magnitude of the angular offset (*δ*) between stripe axis and motion direction is dependent on the direction of rotation and on the orientation of the grating (Fig. 2b(i)–(iii) and Table 1).

**Table 1:** The angular offset between the stripe axis and the motion direction for all three gratings as the drum rotates in the clockwise and anticlockwise directions

Tilt (*φ*)	Angular offset between stripe axis and motion direction (*δ*)	
	Clockwise	Anticlockwise
0°	90°	90°
10°	80°	100°
20°	70°	110°

Because the moving stripes of the rotating gratings provide the main visual feature in the animal’s field of view a strong optical kinetic response is elicited. However, in many animal species the angular offset, δ, between the orientation of the stripe axis and the direction of motion can give rise to ambiguous motion cues due to two visual illusions: (1) the wagon-wheel effect (perceived motion inversion due to spatiotemporal aliasing), and (2) the barber-pole illusion (misperceived direction of motion due to the aperture effect) (How and Zanker 2014). The apparent motion cues generated by the rotation of each orientation of the grating in the clockwise and anticlockwise directions was analysed using the apparent motion algorithm developed by How and Zanker (2014). This algorithm is effectively a Reichardt detector, a theoretical neural circuit comprising a pair of spatial input filters and temporal low-pass filters, connected asymmetrically through a multiplication function (Reichardt 1957, 1961, 1987). The Reichardt detector has been shown to provide a good model approximation for the natural motion detection systems found in a range of animals (Reichardt 1987; Borst and Egelhaaf 1989; How and Zanker 2014).

The actual direction of motion (*ϕ*) of all three grating types is purely horizontal since the gratings are attached to the inner face of the rotating drum;* ϕ* = 90° (clockwise) or* ϕ* = −90° (anticlockwise) (black arrow, Fig. 2c). This is confirmed by the output of the apparent motion algorithm when applied to a simulation of the horizontal movement of the vertical grating (*φ* = 0°); when rotating clockwise the apparent motion of the stripes (blue arrow Fig. 2c(i)) is horizontal and towards the right indicating* ϕ*_A_ = 90° and when rotating anticlockwise, the apparent motion of the stripes (blue arrow Fig. 2c(iv)) is horizontal and towards the left indicating* ϕ*_A_ = −90°. However, when the motion of the tilted gratings (*φ* = 10° or* φ* = 20°) is analysed, there is a component of the apparent motion in the vertical direction. This component is towards the bottom-right of the stimulus during clockwise rotation (as shown by the blue arrows in Fig. 2c(i)(ii)) and towards the upper-left during anticlockwise rotation (as shown by the blue arrows in Fig. 2c(v)(vi)).

### Analysis of the effect of apparent motion

The component of apparent motion in the vertical (pitch) direction, ω_P_, can be approximated using the angular velocity of the drum in the yaw direction (ω_Y_ = 11.63 ± 0.44°s^−1^) and the angular offset, δ, between the orientation of the stripe axes and the direction of motion of the grating;

3 $$\omega_{\text{p}} = \omega_{\text{Y}} { \cos }\left( \delta \right)$$

The values for ω_p_ for each orientation of grating during clockwise and anticlockwise rotation are shown in Table 2.

**Table 2:** The component of apparent motion in the vertical (pitch) direction for each grating orientation during clockwise and anticlockwise rotation

Tilt (*φ*)	Vertical component of apparent motion (ω_P_)/°s^−1^	
	Clockwise	Anticlockwise
0°	0	0
10°	− 2.02	2.02
20°	− 3.98	3.98

The relative speed ratio in pitch, *S*_P_, can be then calculated using *ω*_P_ and measurements of the velocity of eye movements in pitch (i.e., up or down), termed here *eye_pitch_velocity*;

4 $$S_{\text{p}} = \frac{{{\text{eye}}\_{\text{pitch}}\_{\text{velocity}}}}{{\omega_{\text{P}} }}.$$

As for *S*_Y_, S_P_ can be used as a measure of the performance of the eye in matching the apparent motion of the drum. If *S*_P_ = 1, then the pitch velocity (eye_pitch_velocity) of the eye matches the apparent vertical motion (*ω*_P_) of the grating. If S_p_ < 0, the eye is rotating in the opposite direction to the apparent motion, and if S_P_ = 0, the eye is stationary in the pitch degree of rotation. The calculation of S_P_ can only be applied to the rotation of the eye in response to the tilted (*φ* = 10° or *φ* = 20°) gratings since for *φ* = 0°, *ω*_P_ = 0 regardless of the direction of rotation.

### Statistical analyses

All statistical analyses were conducted in R 3.0.25 (R Core Team 2014). The medians and 95% confidence intervals (CIs) were used as measures for non-normal distributions of independent data. Since previous investigations found no significant differences between the rotations of the left and right eyes in response to moving gratings (Daly et al. 2017, 2018), data were pooled across the left and right eyes of each individual. Gaze stabilisation performance was analysed using a generalized linear mixed effects model (GLMM) using the R package lme4 (Bates et al. 2015). The median relative speed ratio (i.e., median *S*_y_) for each trial was calculated across both left and right eyes, the direction of rotation and the trial number were modelled as fixed factors, and individual identity was used as a random term. The level of correlation between torsional rotation and yaw gaze stabilisation performance was investigated using cross-correlation on the differential of the data series with respect to time to satisfy the stationarity assumption (i.e., that there is no overall trend in the data, such that the mean and variance do not change over time) (Chatfield 2004) and to avoid the potential influence of high frequency noise on the correlation calculations. The maximum cross-correlation coefficients were statistically analysed using a Wilcoxon signed rank test to ascertain whether there was evidence of correlations significantly different from zero, which would indicate that the torsional pose of the eye has a significant influence, either positive or negative, on the yaw gaze stabilization response. An additional Wilcoxon test was performed to determine whether the torsional pose of the eye (*θ*_T_), had a significant effect on the median yaw gaze stabilisation performance, as measured by S_y_. In this test torsional pose was categorized as ‘horizontal’ when the midband orientation had an angle (*θ*_T_) with respect to the real-world horizon 0° ≤ *θ*_T_ ≤ 30°, and ‘vertical’ when 60° ≤ *θ*_T_ ≤ 90°. A Wilcoxon test was similarly used to determine whether the median relative pitch ratio (*S*_p_) in response to each of the tilted gratings (*φ* = 10° and *φ* = 20°) was significantly different from 0, which would indicate that the horizontal motion of the tilted gratings does create an apparent motion cue in the vertical direction. Statistical analysis of the effect of the orientation of the grating on the speed of pitch rotation also used a GLMM.

## Results

**Fig. 3:**
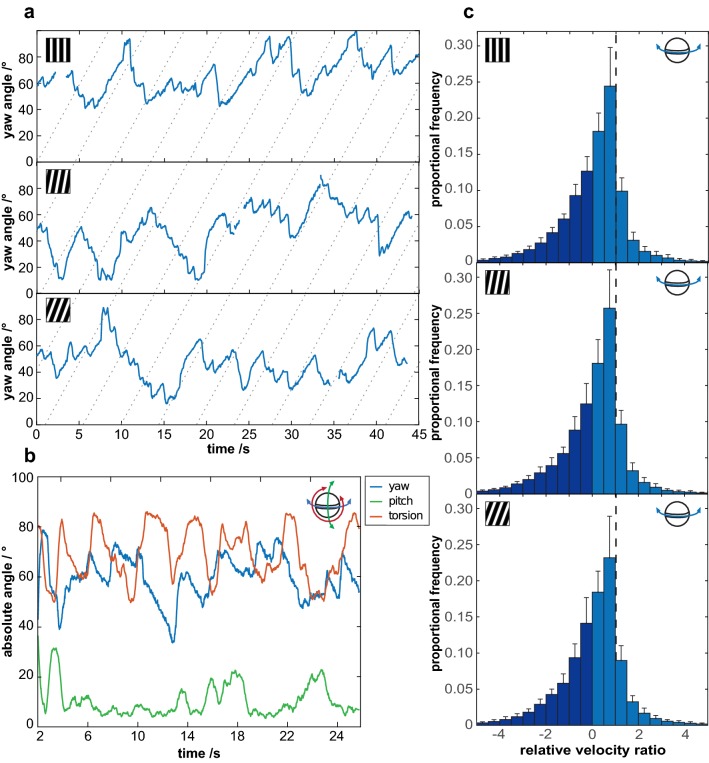
**a** Examples of the stereotypical yaw optokinesis in an eye of three individual stomatopods in response to the motion of the grating rotating in the clockwise direction when the grating was oriented at 0°(top), 10° (middle) and 20° (bottom). The dotted lines indicate the progress of points on the surface of the drum, rotating in the yaw plane, but these lines do not necessarily represent specific stripe boundaries. **b** The yaw optokinesis (blue line) in response to the motion of the grating (*φ* = 20° in this example) is accompanied by pitch (green) and torsion (red) rotations, as demonstrated in the rotation of the left eye of a single individual. **c** The frequency distributions of the median relative velocity ratios recorded in all trials during both the slow and fast phases of yaw optokinesis across both eyes of all six individuals when the grating is oriented at *φ* = 0°(top), *φ* = 10°(middle) and *φ* = 20°(bottom). The dashed vertical lines indicate ‘perfect’, idealized gaze stabilisation (*S*_y_ = 1). *S*_y_ > 0 when the eye is yawing in the same direction as the drum and *S*_y_ < 0 when yawing in the opposite direction (dark blue region), as occurs during fast resets (*n* = 6, error bars are standard deviation across all animals in each 0.5 interval)

*Odontodactylus scyllarus* performed stereotypical yaw optokinesis in response to the horizontal movement of the grating at all three orientations (Fig. 3a). As found in previous work, the animals performed simultaneous pitch and torsion movements (green and red, respectively, in Fig. 3b) as well as yaw optokinesis in response to the horizontal motion of the gratings (Land et al. 1990; Cronin et al. 1991; Daly et al. 2018).

The orientation of the grating (φ) had no significant effect on the gaze stabilization performance (median S_Y_ in the direction of drum rotation, calculated throughout the duration of each trial) (*φ* = 0°: *S*_y_ = 0.74 ± 0.01 [median ± 95% confidence intervals (CI)]; *φ* = 10°: *S*_y_ = 0.72 ± 0.01; *φ* = 20*°: S*_y_ = 0.72 ± 0.02; GLMM, *N *= 6, *χ*^2^ = 1.12, *p *= 0.573; Fig. 3c). The direction of rotation (clockwise or anticlockwise) did not have a significant effect on the gaze stabilization performance either (GLMM, *N *= 6, *χ*^2^ = 0.56, *p *= 0.454). Nor did the trial order (GLMM, *N *= 6, *χ*^2^ = 5.989, *p *= 0.874) have a significant effect. Across the whole distribution of S_Y_, taking the velocity of counter rotations as well as tracking rotations into account, the median of the relative velocity ratios is significantly greater than 0 for all grating orientations (*φ* = 0°: *S*_Y_ = 0.31 ± 0.02 (median ± 95% CI), Wilcoxon sign-ranked test, *N* = 6 V = 21, *p *= 0.0313; *φ* = 10°: *S*_Y_ = 0.31 ± 0.02, Wilcoxon sign-ranked test, *N* = 6 V = 21, *p *= 0.0313; *φ* = 20°: *S*_Y_ = 0.22 ± 0.02, Wilcoxon sign-ranked test, *N* = 6 V = 21, *p *= 0.0313), indicating that the eye movements made by the stomatopods are mostly for gaze stabilization.

**Fig. 4:**
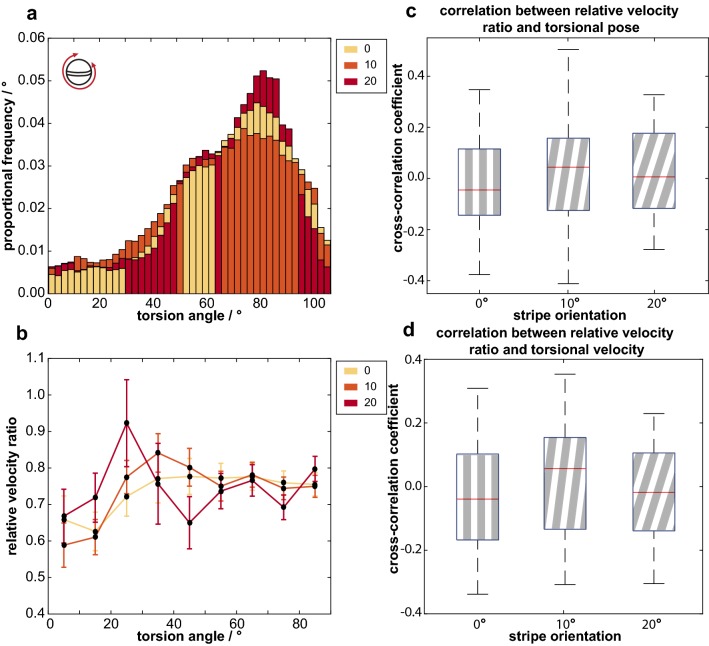
**a** The frequency distributions of the median torsional angles measured in all trials during periods of drum rotation, across both eyes of all six individuals, when the grating is oriented at *φ* = 0° (yellow), *φ* = 10° (orange) and *φ* = 20° (red). **b** Gaze stabilization performance is unaffected by torsion angle at all three grating orientations [*φ* = 0° (yellow), *φ* = 10° (orange) and *φ* = 20° (red)], as shown by the uniform distribution of relative velocity ratios across the range of torsion rotation (*n* = 6, error bars are 95% confidence intervals across all animals in each 10° interval). **c** There is no significant cross-correlation between the angle of torsional pose and relative speed ratio (*S*_Y_) at any grating orientation (*n* = 6). **d** Similarly, there is no significant cross-correlation between the velocity of torsional rotation and the relative speed ratio (*n* = 6)

The orientation of the grating did not have a significant effect on the median torsional pose of the eyes measured as the angle of the midband with respect to the real-world horizon (*φ* = 0° 75.26° ± 0.32° (median ± 95% CI); *φ* = 10° 72.70° ± 0.33°; *φ* = 20° 74.97° ± 0.32°, GLMM*, N *= 6, *  χ*^2^ = 0.457, *p *= 0.796; Fig. 4a). Neither the direction of drum rotation (GLMM*, N *= 6*, χ*^2^ = 0.646, *p *= 0.422) nor the trial number (GLMM*, N * = 6*, χ*^2^ = 4.764, *p *= 0.942) had a significant effect on the torsional pose. As found in previous work (Daly et al. 2018), the torsion angle of the eye did not have a significant effect on the yaw gaze stabilization performance at any grating orientation (cross-correlation coefficient; *φ* = 0°: − 0.02 ± 0.05 (median ± 95% CI), Wilcoxon sign-rank test with a reference value of 0, *N *= 6, *V* = 4, *p *= 0.219; *φ* = 10°: 0.04 ± 0.12, *N *= 6, *V* = 17, *p *= 0.219; *φ* = 20°: 0.02 ± 0.12, *N *= 6, *V* = 16, *p *= 0.313; Fig. 4b, c).

Further to this, the torsional pose of the eye, when divided into the categories ‘horizontal’ (0° ≤ *θ*_T_ ≤ 30°) or ‘vertical’ (60° ≤ *θ*_T_ ≤ 90°), had no significant effect on the median value of *S*_y_ when the eye was oriented in either angular category (*φ* = 0°: *‘*horizontal*’S*_y_ = 0.65 ± 0.03 (median ± 95% CI), ‘vertical’ *S*_y_ = 0.77 ± 0.02, Wilcoxon sign-rank test, *N *= 6, *V* = 6, *p *= 0.438; *φ* = 10°: *‘*horizontal*’ S*_y_ = 0.82 ± 0.03, ‘vertical*’ S*_y_ = 0.74 ± 0.02, Wilcoxon sign-rank test, *N *= 6, *V* = 15, *p *= 0.438; *φ* = 20°: *‘*horizontal*’ S*_y_ = 0.73 ± 0.03, ‘vertical*’ S*_y_ = 0.72 ± 0.02, Wilcoxon sign-rank test, *N *=6, *V* = 12, *p *=0.844). Similarly, the speed of torsional rotation did not have a significant effect on the yaw gaze stabilization performance at any grating orientation (Spearman’s rank correlation coefficient; *φ* = 0°: − 0.03 ± 0.16 (median ± 95% CI), Wilcoxon sign-rank test, *N * = 6, V = 7, *p *=0.563; *φ* = 10°: 0.06 ± 0.16, *N *= 6, *V* = 16, *p *= 0.313; *φ* = 20°: − 0.02 ± 0.12, *N *= 6, *V* = 6, *p *= 0.438; Fig. 4d).

**Fig. 5:**
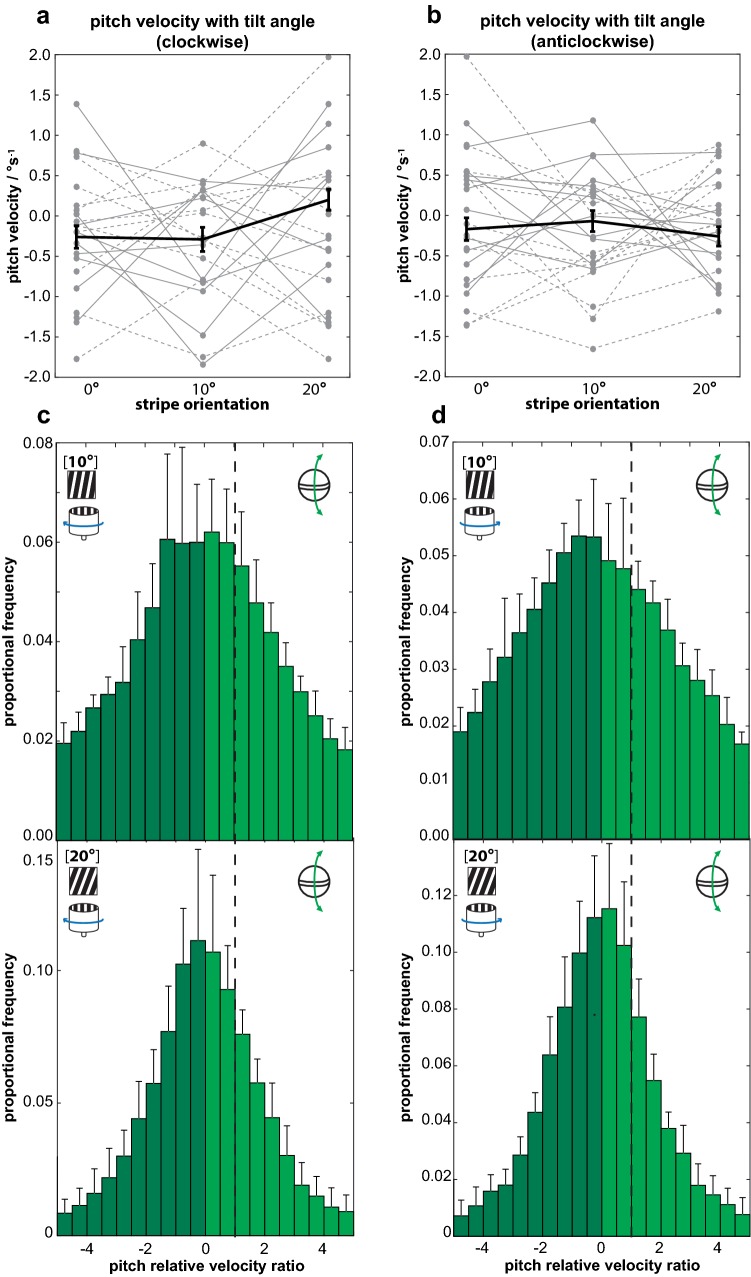
**a** The velocity of the pitch rotations shows no strong dependence on stripe orientation when the drum is rotating in the clockwise direction. Median pitch velocity across all animals shown in black, with the responses of the left (solid grey) and right (dashed grey) eye of each individual across all trials (*n* = 6, error bars are 95% confidence intervals across all animals in each 10° interval). **b** The median distribution of the pitch relative velocity during clockwise rotation of the drum when the grating is oriented at *φ* = 10° (top) and *φ* = 20° (bottom). Dashed vertical line indicates ‘perfect’, idealized gaze stabilisation (*S*_P_ = 1). *S*_P_ > 0 when the eye is pitching in the same direction as the apparent motion of the grating and *S*_P_ < 0 when yawing in the opposite direction (dark green region) (*n* = 6, error bars are standard deviation across all animals in each 0.5 interval). **c**, **d** Similar results are seen in response to anticlockwise rotation of the drum. The similarity in the overall distribution of the pitch speed in response to clockwise and anticlockwise rotation indicates that the mantis shrimp visual system is unaffected by the apparent downward (clockwise rotation) or upward (anticlockwise) motion generated by the horizontal movement of the tilted gratings. Note that the relative differences in the width of the distributions of S_P_ in response to gratings oriented at 10° and 20° is due to the calculation of S_P_; the magnitude of the vertical component of apparent motion (*ω*_P_, Eq 2, Table 2) is greater for 20° than for 10°. Since the values of the pitch speed **a**, **c** are not significantly different, when the 20 data is divided by ω_P_, the resulting distribution is smaller, with a higher proportion of the S_P_ data in the bins closest to 0

There were no instances in any of the trials in which the pitch rotation of the eye appeared to be tracking the motion of the drum either downwards or upwards, as would be expected if apparent motion artefacts had an affect on perceived grating movement (see Fig. 2c). The speed of pitch rotation was not significantly affected by the orientation of the gratings (GLMM*, N *=* 6, χ*^2^ = 5.04, *p *= 0.081; Fig. 5) nor the trial number (GLMM*, N *=* 6, χ*^2^ = 15.57, *p *= 0.158). Despite the prediction that the clockwise rotation of the tilted gratings would cause the eyes to pitch downwards and the anticlockwise rotation to cause them to pitch upwards, the rotation direction had no significant effect on the speed of pitch rotations (*φ* = 0°: clockwise − 0.25 ± 0.15°s^−1^, anticlockwise − 0.17 ± 0.14°s^−1^; *φ* = 10°: clockwise − 0.29 ± 0.15°s^−1^, anticlockwise − 0.07 ± 0.13°s^−1^; *φ* = 20°: clockwise 0.20 ± 0.14°s^−1^, anticlockwise − 0.26 ± 0.12°s^−1^; GLMM*, N *=* 6, χ*^2^ = 0.27, *p *=0.607; Fig. 5a, b). Following this, an additional analysis determined that the pitch relative velocity ratios (*S*_p_) of the eyes in response to the tilted (*φ* = 10° and *φ* = 20°) gratings were not significantly different from zero (*φ* = 10°: clockwise *S*_P_ = − 0.03 ± 0.61, anticlockwise *S*_P=_0.00 ± 0.36; *N* = 6, *V* = 11.0, *p *= 1; Wilcoxon sign-ranked test*, N* = 6, *V* = 19.5, *p *= 0.074; *φ* = 20°: clockwise *S*_P _= − 0.05 ± 0.61; *N* = 6, *V* = 15.5, *p *= 0.344, anticlockwise *S*_P _= 0.01 ± 0.20; *N* = 6, *V* = 4.5, *p *= 0.24; Fig. 5c, d). This indicates that the eyes are not following any vertical motion cues caused by visual illusions arising from the offset of the tilted gratings and the actual direction of motion.

## Discussion

The results of this somewhat unusual version of a gaze stabilization experiment demonstrate that, irrespective of the speed, direction and angular offset of a motion stimulus and the torsional pose of the eye, mantis shrimp perform yaw gaze stabilization equally well. This offers additional evidence in support of our prior conclusion that the neural wide-field motion detection network in the stomatopod visual system is robust to apparent movement and may, therefore, follow a radially symmetric organization (Daly et al. 2018). The evidence to support this theory is now threefold: firstly, the gaze stabilization performance is highly attuned to actual movement and is unaffected by the ambiguous motion cues generated by the offset between the long axis of the stripes and the direction of motion; secondly, it is similarly unaffected by the torsional rotation of the eyes; and finally, there is no evidence of torsional gaze stabilization (Daly et al. 2018). Such evidence strongly suggests that the neuronal network for detecting wide-field motion in the stomatopod eye must be more complex than a simple system of a Reichardt-like motion detector involving a comparison between the outputs of pairs of photoreceptors that are spatially separated vertically or horizontally in the retina (or in adjacent ommatidia across a compound eye) (Reichardt 1957, 1961, 1987). The gaze stabilization response of many insects originates with directionally selective wide-field neurons that have a specific orientation in the eye relative to real-world coordinates (e.g., (Franz and Krapp 2000; Borst and Haag 2002)). In contrast, gaze-stabilizing mechanisms in stomatopods, with their torsionally rotating eyes, would likely require a different architecture that is optimized to a shifting coordinate system given the wide range of torsional rotational freedom of their eyes.

One possible set of candidates for the neurons underlying such a rotationally insensitive motion detector are the ‘space-constant fibres’. These neurons, first identified in crayfish (Wiersma and Yamaguchi 1966) and subsequently found in rock lobsters (Wiersma 1967; Wiersma and Yanagisawa 1971) and crabs (Wiersma 1970; Wiersma et al. 1977) have a potential receptive field that covers the entire retina, but the actual receptive field changes with the position of the animal. For instance, in the crayfish *Procambarus clarkii*, Wiersma and Yamaguchi (1966) recorded from a fibre that was sensitive to a moving light stimulus only in the dorsal part of the eye and not the ventral. However, when the animal was turned upside down, the fibre carried signals of responses only from the ventral (now uppermost; above the horizon) and not the dorsal (now below the horizon) part of the eye. There is evidence that the function of these ‘space-constant’ fibres depend on input from the statocysts and proprioceptive signals from leg joints (Wiersma et al. 1982). While stomatopods do not have statocysts, there is likely to be some form of positional information either from the eight muscles controlling eye movement (Jones 1994), the leg joints or possibly the position of the antenules which are known to have proprioceptive organs (Sandeman 1964). While there is a strong possibility that some form of these ‘space-constant’ fibres underlies the torsionally invariant motion detection circuitry, they have yet to be identified in the stomatopod visual system. Further electrophysiological studies along with psychophysical behavioural experiments are needed to test this theory.

Despite the tilted gratings (angular offsets of 10° and 20°) having an apparent component of motion in the vertical direction, we found no statistical difference in the pitch responses to all three gratings when the drum was rotating in either direction. Given the level of apparent motion generated by the torsional rotation of the eyes, it is perhaps unsurprising that the motion detection in the stomatopod visual system is seemingly robust to this type of apparent motion. However, it is possible that there was a differential pitch response to the three orientations of grating, but that its magnitude was insufficient in comparison with the background levels of pitch rotation for the response to show as significant. The effect may further have been masked by the relatively small sample size. An additional consideration surrounding the lack of a strong pitch response is the inequality in the speeds of the two components of the motion; the actual horizontal stimulus had a speed of approx. 12°s^−1^, while the speed of the apparent vertical motion was far more modest (~ 3°s^−1^ or 4°s^−1^ depending on the grating orientation). Add to this that, due to the grating covering the entire inner face of the cylindrical rotating drum, the horizontal stimulus filled the entire horizontal (yaw) field of view, while the apparent vertical motion filled only part of the vertical (pitch) field of view. Consequently, the full-field horizontal motion should drive a strong response from the motion-sensitive neurons tuned to yaw rotation, while the apparent vertical motion is only likely to drive a weak response from neurons tuned to pitch rotation. Additionally, we cannot discount the possibility that the top and bottom edges of the stimulus acted as a fixed ‘horizon’, allowing for the animals to disambiguate the actual from the apparent motion cues arising from the aperture effect. These uncertainties may be addressed in future work using gratings with more extreme angular offsets and attempting to reduce the effect of fixed horizons (such as the top and bottom of the stimulus) perhaps using a screen to display a virtual grating with blurred edges such that there is no sharp transition to act as a ‘horizon’.

The torsional pose of the eyes was unaffected by variation in the orientation of the major axes of the primary features of the scene (i.e., the orientation of the grating), indicating that the torsional pose of the eye is independent of the features of a striped pattern that dominated the visual scene in our experiments and in which the stimulus was monochrome and relatively simplistic. However, the amount of torsional rotation of the stomatopods’ eyes observed during drum rotation was highly variable, exhibiting oscillations, random rotations, and stable poses (Fig. 3b, Fig. 4a). It is thus possible that there was some difference in the torsion angle with the grating orientation, but that the large range of torsional rotation masked its effect. Additionally, the stomatopods were given 3–4 min to habituate to the appearance of the stationary drum before drum rotation started and the eye tracking began. It is, therefore, possible that there is some initial relationship between feature orientation (i.e., stationary grating stripe angle) and torsional pose when a scene is first presented but we did not attempt to investigate this idea. While torsional rotations have been shown to play a role in stomatopod dynamic polarization vision (Daly et al. 2016), their full function has yet to be satisfactorily explained. Due to their colour receptors being restricted to the midband region of their compound eyes, effectively resulting in one-dimensional colour vision (Marshall et al. 1991b), stomatopods ‘scan’ their eyes across the visual scene (in particular, moving them in a direction orthogonal to the midband) to obtain colour information from objects (Land et al. 1990). Future work should examine the effect of the spectral and temporal diversity of a visual scene on the torsional pose and speed of torsional rotation during such scans.

Future work should also determine whether the apparent lack of any effect of ocular torsional rotational on the stomatopod’s motion detection system holds during tracking of smaller objects, or objects following a complicated trajectory. *Odontodactylus scyllarus* does not exhibit a strong tracking response to objects that subtend only a small visual angle (Daly, personal observation) but other mantis shrimp species, such as *Gonodactylus oerstedii,* show strong visual fixation and tracking (Cronin et al. 1988). If wide-field stimulus tracking of a high contrast grating is also demonstrated in these species, and is shown to be unaffected by ocular torsion, future investigations should focus on the accuracy of small-object tracking as a function of torsional eye rotation. This assumes, however, that such torsional rotations are actually performed during small-object tracking and it is possible that a greater degree of torsional stabilization may be required during tasks involving more complex motion.

Finally, it must also be noted that visual cues other than the information present in the immediate frontal field of view may be playing a role in the vertical gaze stabilization of mantis shrimp. For instance, the position of lighting within an experimental setup was shown to have a strong influence on the ability of hoverflies to stabilize their flight, even in the presence of visual stimuli on the walls of the arena (Goulard et al. 2018). Stabilization performance was better when the experimental arena was illuminated from above than when it was illuminated from below. In almost all naturalistic conditions, the down-welling light originating from the sun will be the predominant light source in the natural environment of *O. scyllarus* even at the maximum extremity of its depth range (ca. 40 m). It is possible that stomatopods have a similar dorsal light response (DLR) to hoverflies which, given that illumination in this set of experiments was from above, might also account for their ability to distinguish between real and apparent vertical motion cues. However, the lack of stabilization in the torsional degree of freedom in the stomatopod visual system (Daly et al. 2018) adds another layer of complication in comparison to the visual system of animals, such as hoverflies, which have a highly stabilized gaze (Collett and Land 1975; Land 1999; Goulard et al. 2015). For instance, when a stomatopod has an eye oriented such that the midband is horizontal, the dorsal region of the eye will experience higher illumination than the ventral region. However, if the eye rotates torsionally through 90°, such that the midband is now vertical, both dorsal and ventral regions will experience the same level of illumination. It is possible that there is a ring of ommatidia distributed about the periphery of the entire eye that may be responsible for a potential DLR. Indeed, detection of the direction of the down-welling light could be one mechanism by which the stomatopod visual system compensates for the potentially disorientating effects of torsional rotation. Future investigations should focus on the influence the environmental light conditions, such as the direction of illumination, have on the stomatopod gaze stabilization response.

## Conclusion

In conclusion, we find further evidence that the stomatopod visual system is unaffected by any potentially image degrading effects due to torsional self-motion and appears robust to ambiguous ‘apparent motion’ cues that are a feature of vision in other animals, including humans. Understanding how stomatopods do this could be valuable for any robotic vision system that needs to maintain visual stability, or track objects, whilst moving with complex or unpredictable trajectories.
